# Development of Preschoolers’ Understanding of Zero

**DOI:** 10.3389/fpsyg.2021.583734

**Published:** 2021-07-27

**Authors:** Attila Krajcsi, Petia Kojouharova, Gábor Lengyel

**Affiliations:** ^1^Cognitive Psychology Department, Institute of Psychology, ELTE Eötvös Loránd University, Budapest, Hungary; ^2^Doctoral School of Psychology, ELTE Eötvös Loránd University, Budapest, Hungary; ^3^Institute of Cognitive Neuroscience and Psychology, Research Centre for Natural Sciences, Budapest, Hungary; ^4^Department of Cognitive Science, Central European University, Budapest, Hungary

**Keywords:** numerical cognition, zero, number status of zero, items based number representation, cardinality principle

## Abstract

While knowledge on the development of understanding positive integers is rapidly growing, the development of understanding zero remains not well-understood. Here, we test several components of preschoolers’ understanding of zero: Whether they can use empty sets in numerical tasks (as measured with comparison, addition, and subtraction tasks); whether they can use empty sets soon after they understand the cardinality principle (cardinality-principle knowledge is measured with the give-N task); whether they know what the word “zero” refers to (tested in all tasks in this study); and whether they categorize zero as a number (as measured with the smallest-number and is-it-a-number tasks). The results show that preschoolers can handle empty sets in numerical tasks as soon as they can handle positive numbers and as soon as, or even earlier than, they understand the cardinality principle. Some also know that these sets are labeled as “zero.” However, preschoolers are unsure whether zero is a number. These results identify three components of knowledge about zero: operational knowledge, linguistic knowledge, and meta-knowledge. To account for these results, we propose that preschoolers may understand numbers as the properties of items or objects in a set. In this view, zero is not regarded as a number because an empty set does not include any items, and missing items cannot have any properties, therefore, they cannot have the number property either. This model can explain why zero is handled correctly in numerical tasks even though it is not regarded as a number.

## Highlights

- Preschoolers can handle zero as soon as they can handle positive integers.
- Preschoolers are unsure whether zero is a number.
- Children may start to understand numbers as the properties of items in a set.

## Introduction

Children start to understand the use of symbolic exact numbers at around the age of three (Wynn, 1990, 1992). Although many details on the development of understanding natural numbers are already known, the development of understanding zero remains mostly unknown, and it is not integrated into any numerical cognition models. It is still largely unknown how zero is handled and understood in tasks in which symbolic natural numbers are already used successfully by preschoolers. The main aim of the present study is to describe more fully the development of understanding zero and to consider its theoretical implications.

### Lack of Developmental Models for Understanding Zero

Models concerning the development of numerical cognition mostly cannot specify how understanding zero is integrated into more general numerical knowledge. As a starting point, in the infant literature, there is agreement that non-symbolic numerical information (such as arrays of dots, and series of sounds or events) is probably processed by two representations (Feigenson et al., 2004; Piazza, 2010). Dominant models propose that, in infants, numerical information is handled by either the imprecise Approximate Number System (Feigenson et al., 2004; Piazza, 2010) or the visual attention related Object Tracking System (Feigenson et al., 2004). However, it is not straightforward whether either of these systems can handle zero (see more details on how these models may or may not account for zero processing in Supplementary Material).

The next important step in the development of number understanding is for preschoolers to acquire an understanding of exact large symbolic numbers. Symbolic numbers are values that are denoted by symbols (in the case of preschoolers, such symbols are usually number words), as opposed to non-symbolic quantities, such as arrays of visual objects or series of auditory events. According to the consensus in the literature, at around the age of three or four, children start to understand the conceptual principles of number use, which is referred to as understanding the cardinality principle (Wynn, 1990; Lipton and Spelke, 2006; Sarnecka and Carey, 2008). With this principle, they are able to handle exact symbolic numbers. Number knowledge in preschoolers is usually measured with the “give a number” (or give-N) task, in which children are asked to give a specific number of objects from a pile of objects (Wynn, 1990, 1992). With this task, one can determine what phase of number understanding a child is in. The first phase is the pre-numeric phase; in this phase, although preschoolers know the counting list (i.e., the series of number words starting with “one-two-three”), they do not know the meaning of these words and fail in the task. These children are termed pre-knowers. The second phase is when children become subset-knowers; they can give 1, 2, 3, or 4 items, but not when asked for more, even when they know how the counting list continues. The final phase is when preschoolers become cardinality-principle-knowers (CP-knowers); they can give any amount of items that is in their known counting list. This phase is believed to show their real understanding of exact symbolic numbers (Wynn, 1990, 1992; Lipton and Spelke, 2006; Sarnecka and Carey, 2008). (Although see some limitations of this description, for example, in Le Corre and Carey, 2007; Davidson et al., 2012; Le Corre, 2014; Sella and Lucangeli, 2020).

There is no consensus on what representational changes occur for a preschooler to understand the cardinality principle. Most models suppose that, in some way, the systems available in infancy may play a role in the first steps (Carey, 2004, 2009; Piazza, 2010), although it is not known entirely how these or other systems contribute to their understanding of symbolic exact numbers. Relatedly, it is not known whether preschoolers understand symbolic zero when they understand the cardinality principle. Importantly, the few works that have investigated preschoolers’ symbolic understanding of zero (see the following subsection) cannot be integrated into this framework because those works did not investigate whether or not the preschoolers understood the cardinality principle.

### Contradictory Results on Preschoolers’ Understanding of Zero

To our knowledge, there are only two studies on preschoolers investigating quantitatively the zero concept when it is denoted symbolically (Wellman and Miller, 1986; Bialystok and Codd, 2000). Note again that the present work focuses on the processing of symbolic stimuli because (a) investigating non-symbolic zero involves many unresolved methodological issues, (b) in recent years, several works have revealed essential differences between symbolic and non-symbolic number processing (e.g., Noël and Rousselle, 2011; Bulthé et al., 2015; Krajcsi et al., 2016, 2020; Schneider et al., 2017), and such differences put into question whether symbolic stimuli are processed by an evolutionarily old, imprecise number representation, and (c) CP-knowledge points beyond approximate number handling (Carey and Barner, 2019)^1^. As mentioned above, neither of these two studies is related to the current developmental models. Importantly, the conclusions of these two works contradict each other on whether or not handling zero is more difficult for preschoolers compared to handling positive integers. In this subsection, we briefly summarize the two studies, and then discuss the potential causes of their differing conclusions.

Wellman and Miller (1986) presented children between 3 and 7 years old with the following tasks: (a) count items in sets (including empty sets), (b) name the smallest number they know, (c) name Indo-Arabic symbols, and (d) compare numbers between 0 and 5 in Indo-Arabic notation. Their main finding was that the children’s understanding and use of zero were delayed compared to their use of positive numbers. In their detailed analysis, the authors concluded that there are three typical behavioral patterns or, in their terminology, phases. In the first phase, children can name the 0 symbol, although they do not understand its meaning. In the second phase, children can count backward to zero, with the understanding that zero means nothing. Finally, in the third phase, children know that zero is the smallest number, and they can compare numbers even if one of the numbers is zero. In this description, the progress of their development is slow: 4-year-old children are not yet at the first phase, and only 6-year-old children are at the final phase.

However, a later study found evidence that understanding zero may be as uncomplicated as positive numbers for preschoolers. Bialystok and Codd (2000) investigated preschoolers’ understanding and spontaneous notation of positive integers, zero, and fractions. They found that, for preschoolers, understanding zero is not harder than understanding positive integers; this conclusion is not in line with the previously described study by Wellman and Miller (1986). In this work, children between 3 and 7 years old were asked to give different amounts of cookies to puppets and to make written notes about these amounts. The children had to recall the amounts 20 min later; and again 2 weeks later. In both instances, the children were allowed to use the notes they had made. According to the results, the children were able to solve the give-zero task. However, it is important to note that the instruction was not formed in the usual mathematical way, e.g., the children were not told to “give Big Bird zero cookies”; instead they were instructed to “give Big Bird no cookies for lunch.” The children were able to make a note of the number zero as efficiently as making a note of positive integers. Similarly, they could recall the correct number after 20 min, be it zero or a positive integer. However, 2 weeks later, unlike 5-year-old children, 3- and 4-year-old children were unable to recall the number zero as successfully as recalling positive integers. (The same result was found by Hughes, 1986, who found that children can use notes for denoting zero, however, the quantitative results of that study were not published).

The contradiction between the conclusions of these two studies (i.e., Wellman and Miller, 1986; Bialystok and Codd, 2000) may originate from methodological differences and from interpretational issues. Obviously, the two studies used completely different tasks, and it is possible that zero can be handled more easily in some tasks than in others. Yet, there are some less trivial sources of differences. First, while Wellman and Miller (1986) suggest three phases of development for understanding zero, their data actually seem to reveal four phases. (Even though the authors mentioned that the data may include some inconsistencies, they nonetheless insisted on an interpretation with three phases.) The additional phase is between the second and third phases: After successfully counting back to zero, the preschoolers could compare numbers with zero, even though they did not know that zero is the smallest number (see Table 1 in Wellman and Miller, 1986). In fact, this phase is paradoxical: While children know that zero is smaller than one, they think that one is the smallest number. What causes this dissociation in their zero-knowledge? As a possible explanation, we hypothesize that children do not think that zero is a number. This possible misconception is even observable in adults: In a study, 15% of preservice elementary school teachers responded that zero is not a number (Wheeler and Feghali, 1983). Another possible explanation is that zero is not part of the counting list (which usually starts with “one”), and this is why children handle zero differently (Merritt and Brannon, 2013). Both explanations suggest that their meta-knowledge of the number status of zero may be independent of handling the zero value correctly. Consequently, one may assume that children can understand zero sufficiently when they compare zero correctly, but they do not yet understand that zero should be categorized as a number. If this is the case, children may understand zero earlier than what was proposed by Wellman and Miller. We return to this problem and to a more detailed list of possible explanations in the discussion section.

A second methodological problem that could be the cause of the two studies’ different conclusions is that the linguistic formulation of the tasks including zero could have influenced performance. While the mathematical viewpoint suggests that zero is a number just like any other integer and that zero should therefore be used linguistically in the same way as other numbers, natural language mostly uses different linguistic forms for statements about zero. For example, we usually do not say “The car is traveling with zero kilometers per hour”; instead, we say “The car has stopped (or is stationary).” Similarly, we do not say “Give zero cookies to Peppa Pig”; instead, we say “Do not give any cookies to Peppa Pig” or “Give no cookies to Peppa Pig.” Thus, it is possible that using mathematical language is harder for children than using natural language because the former is less familiar to them. This can be hypothetically confirmed by the data from the two studies: The Wellman and Miller (1986) used mathematical language and found that children experienced difficulties in understanding zero, while Bialystok and Codd (2000) used natural language and found that children experienced no difficulties in handling zero. However, based only on these data, one cannot be sure whether language form significantly influenced performance, because of the many other differences between the two studies.

### Aims of the Study

To create appropriate models, it is essential to first have reliable data. Considering that the two previously discussed studies came to contradictory conclusions on whether processing the zero value compared to processing positive values is harder for preschoolers (Wellman and Miller, 1986; Bialystok and Codd, 2000), and that we cannot confirm the causes of the contradictions relying on the two studies’ data, the present study aims to provide additional more systematically collected data on preschoolers’ symbolic understanding of zero. Note that this means that our aims do not build upon any theoretical models, such as the Approximate Number System, or the Object Tracking System (presented in the first part of the Introduction), rather, this study seeks to clarify the main phenomena and describe the development more precisely and extensively than previous works have (presented in the second part of the Introduction).

(Aim 1) Specifically, to use a more comprehensive range of tasks (i.e., giving a set, comparison, addition and subtraction) in order to investigate whether children can handle zero in numerical tasks as efficiently as they can handle positive integers (Aim 2). To test the potential effect of language form (contrasting mathematical vs. natural language and investigating whether the number word “zero” is understood) on performance in the tasks (Aim 3). To put these findings into the context of current models for understanding the cardinality principle, the present study investigates whether subset-knowers and cardinality-principle-knowers (as measured with the give-N task) have a different level of understanding of zero (if understanding zero is available at such an early age) (Aim 4). To investigate whether the additional phase we emphasized in the data from Wellman and Miller (1986) is reliably observable, i.e., whether at some point children can compare zero correctly, even though they still think that zero is not a number.

## Materials and Methods

### Participants

Forty 3- and 4-year-old Hungarian preschoolers participated in this study. Because of the methodological limitation of the applied give-N task, the number knowledge of two of these children (i.e., whether they were one-knowers or pre-knowers) could not be specified; consequently, their data were excluded from the study (find more details in the tasks and results sections). The data of 19 boys and 19 girls were analyzed, with a mean age of 4 years and 1 months, ranging between 3 years 2 months and 5 years 1 month. Two preschools were involved in the data collection; one of them in the capital and the other one in a country town (18 preschoolers from the capital, 7 girls in the capital and 12 girls in the country town). The children in both preschools were mostly from middle-class families. None of the preschoolers had previously received formal training on handling zero in preschools. No further data were collected on their sociodemographic characteristics or former numerical training.

### Tasks

The tasks used in this study covered three main areas (Table 1). Note that the present tasks mainly investigate the handling of symbolic numbers. (1) The give-N task categorized children in terms of whether they were cardinality-principle-knowers (CP-knowers) or subset-knowers. (2) This task was also used with “zero” and “nothing” to determine whether the preschoolers can apply the zero value in the task. Additionally, comparison, addition, and subtraction tasks were used to measure whether the preschoolers could use zero in operations as efficiently as they could use positive integers. These tasks were also used to measure the effect of various linguistic versions of the same tasks. (3) The preschoolers’ meta-knowledge of numbers was measured to investigate whether they understood that zero is a number.

**TABLE 1:** Summary of the tasks.

Name of the task	Short description
**Measuring number knowledge**	
Give-N (positive numbers)	Give N balls to an agent.
**Operations with zero**	
Give-N (nothing and zero)	Give N balls to an agent.
Comparison	Choose the larger set.
Addition	Add two values.
Subtraction	Subtract one value from another.
**Meta-knowledge of zero**	
Smallest number	Name the smallest number.
Is it a number?	Say whether something is a number or not.

#### Tasks Related to the Aims

Because the present study includes four independent aims (independent in the sense that any of the aims would be meaningful without the others), and because many of the tasks contribute to several of the aims at the same time, we give explicit guidance throughout the text on what aspects of the tasks or task-combinations contribute to which aims. Here, we review the aims and what aspects of the tasks investigate those aims. Aim 1 (is zero more difficult to handle than positive integers for preschoolers) is measured with the operations tasks. The relevant contrast is whether zero-related operations compared to positive number-related operations show worse performance. Aim 2 (the role of the linguistic form in understanding zero) is measured across all tasks: The give-N task measures the preschoolers’ interpretation of the “zero” and “nothing” labels in that task; the operations tasks measure the effect of the mathematical and natural linguistic forms; and the meta-knowledge tasks investigate again the “zero” and “nothing” labels in those contexts. The relevant contrast is whether different linguistic versions induce different levels of performance. Aim 3 (the role of number knowledge in terms of subset-knowers and CP-knowers) is measured in both the operations and meta-knowledge tasks by contrasting the two number-knowledge groups. Finally, Aim 4 (do preschoolers lack meta-knowledge about zero when they can handle zero in operations) is investigated in the meta-knowledge tasks, whose results are contrasted with the results of the operations tasks (See also the analysis plan below).

#### Give-N Task

In this task a pile of balls was in view of the children, and they had to give a specific number of balls to a toy bird. The task measures (a) whether the child understands the cardinality principle, and (b) whether the child understands what the words “nothing” and/or “zero” refer to.

Note that while the performance with positive numbers has been investigated and validated extensively (for example see the seminal works of Wynn, 1990, 1992), to our knowledge, performance with zero in this task has not been studied yet. Also, to our knowledge, understanding zero and understanding positive numbers has not been measured together in the give-N task, and theoretically, it is possible that they are independent (i.e., knowledge on positive numbers in itself cannot predict zero-knowledge). Importantly, in the present study, zero-knowledge is measured with the same task and with the same criteria as positive integer-knowledge; thus, this method could serve as an appropriate starting point to categorize the children in terms of whether they know what “zero” refers to. The give-N task used in this study is similar to the give-a-number task as described by Wynn (1990) and Bialystok and Codd (2000), which is the consensually accepted tool to measure preschoolers’ cardinality principle- and number-knowledge. At the end of the trial, the experimenter explicitly asked the child whether they were done with the task. This is essential in tasks including zero-trials since the child does not have to give any items.

When measuring their understanding of zero with the task, two versions of zero were applied. For the “natural” version of zero, we utilized the form that is used in everyday language: “Do not give any balls to the bird.” For the “mathematical” version, we used the form that reflects the mathematical viewpoint: “Give zero balls to the bird.” In Hungarian, nouns after a number word are always singular, e.g., “give zero *ball*” or “give two *ball*,” therefore, plural vs. singular form did not influence how the children solved the task (See the questions in all tasks in Hungarian and in their English translations in Table A1).

Overall, the following six numbers were tested in the given order: 2, 0 (natural), 5, 3, 0 (mathematical), 4. The present order of the numbers was used to prevent the children from relying on an order of increasing number words in the task. The whole number series was repeated twice, resulting in 12 trials.

It has been argued that children solving tasks with numbers 4 or larger are exhibiting an understanding of the cardinality principle (Wynn, 1990, 1992; Condry and Spelke, 2008; Sarnecka and Carey, 2008). In this study, a child was categorized as knowing a number if both trials of that number were solved correctly and if known numbers were not given as a response to higher unknown numbers^2^. Following these results, children were categorized as CP-knowers if they could give the numbers 4 and 5 in both trials; otherwise, they were categorized as subset-knowers. Categorization based on this task was performed right after the child completed the task; this was done because the comparison, addition, and subtraction task stimuli (see below) depended on the children’s number knowledge.

Independently of the previous categorization, children were categorized as “nothing-givers” if they correctly did not give anything in the natural-zero task in both trials and as “zero-givers” if they correctly did not give anything in the mathematical-zero tasks in both trials. (Note that, because the performance of the give-N task with positive numbers is well-known in the literature, children who successfully give a specific value are termed “knowers,” such as one-knowers, subset-knowers, or CP-knowers. However, such knowledge in the literature is not available for zero; therefore, to highlight the fact that it is not clear whether children solving this task with zero really understand some key features of zero, we term such children as nothing-”givers” or zero-”givers,” instead of using the term “knowers,” i.e., we are referring to the performance in the task instead of the supposed knowledge of the child).

#### Comparison

The aim of this task was to test whether the children knew the position of zero among other numbers, therefore, whether they are able to handle zero as efficiently as positive numbers (Aim 1) and to investigate whether the form of the task (natural verbal vs. mathematical verbal vs. non-verbal) influences their performance (Aim 2). Additionally, the number-knowledge groups, as identified by the give-N task, are compared in the task (Aim 3), and comparison operation performance is contrasted with the meta-knowledge tasks (Aim 4). In the task, the children either saw two sets of objects or heard two numbers, and had to choose the larger one.

To test whether the children understood the verbal description of the task, we used both verbal and a non-verbal object versions. In the object version, two sets of balls were placed on opposite sides of a table, and the question was, “On which side can you see more?” The number of the objects in a set was not named by the experimenter. In the case of the zero value, the appropriate side of the table remained empty. In the verbal version, no objects were used, and the question was, “Which one is more, the x or the y?” (The Hungarian translation of “which one” does not include the word “one,” thus, this part of the question would not have confused the children.) In the verbal condition, the number zero was labeled as either “zero” (mathematical version) or “nothing” (natural version).

If the children understood the cardinality principle as measured by the give-N task, the following 12 number pairs were tested in the given order: 3–2, 4–1, 2–4, 3–zero, 2-nothing, 1–5, zero–4, 1–3, 2-zero, nothing-4, 2–1, and zero–1 (4 pairs with “zero,” 2 pairs with “nothing,” and 6 pairs with only positive values). Otherwise, the following 10 number pairs were tested in the given order: 3–2, 2–1, 1–2, 1–zero, 2–nothing, 1–3, zero–2, 1–2, 2–zero, and nothing-1 (3 pairs with “zero,” 2 pairs with “nothing,” and 5 pairs with only positive values). The use of the two series for the two groups ensures that (a) the children see tasks only with a number range corresponding to their capability, while (b) it is possible to measure a relatively wide number range in the CP-knower group. Note that the two series do not prevent us from investigating the main questions: whether zero is processed as correctly as positive numbers and whether linguistic forms have an effect on performance, because no direct comparison of the two groups in terms of positive number performance is required, and only tasks or conditions within the groups are compared. Because, in the object condition, the “zero” and “nothing” versions mean the same stimulus (i.e., missing objects), only trials with “zero” were tested.

In the analyses, the percentage of correct responses in the “zero,” “nothing,” and positive trials was used as the task’s performance index.

#### Addition and Subtraction

As in the comparison task, the aim for the addition and subtraction tasks was to test whether children can handle zero as efficiently as positive numbers in arithmetic operations (Aim 1), whether the form of the task (natural verbal vs. mathematical verbal vs. non-verbal) influences their performance (Aim 2), whether the number-knowledge groups as identified by the give-N task perform differently in the arithmetic tasks (Aim 3), and whether zero handling in arithmetic task performance is better than meta-knowledge task performance (Aim 4). In the arithmetic tasks, the children had to add or subtract two numbers and say the result.

To test whether the children understood the verbal description of the task, the tasks were either in verbal form or shown with objects. In the verbal form, either the “natural” or “mathematical” form of zero was used. In the object version, while the task was also explained in verbal form (in the case of zero, both in the “natural” and “mathematical” forms), the operands of the task were shown with arrays of balls: one operand on one side and the other operand on the other side of the table. In the case of zero, the appropriate side of the table remained empty.

If the child understood the cardinality principle as measured by the give-N task, the following 15 operations were tested in the given order: 1 + 1, 3 + 1, 2 + 0, 1 + 2, 0 + 3, 2 + nothing, nothing + 3; 2–1, 4–2, 3–0, 3–1, 2–0, 2–2, 3–nothing, and 2-nothing (4 tasks with “nothing,” 4 tasks with 0, and 7 tasks with only positive values). Otherwise, the following 13 operations were tested in the given order:1 + 1, 2 + 1, 2 + 0, 1 + 2, 0 + 1, 2 + nothing, nothing + 1; 2–1, 2–0, 1–0, 1–1, 2–nothing, and 1-nothing (4 tasks with “nothing,” 4 tasks with 0, and 5 tasks with only positive values only). The motivation for using the two series was the same as in the comparison task.

The task was embedded in a small story. In the addition task, the following story was told to the children: “The bird had x balls. The dog had y balls. How many balls do they have altogether when they play together?” In the subtraction task, the story was as follows: “The bird had x balls. Then, the bird gave y balls to the dog as a present. How many balls is the bird left with?” In the “natural” linguistic version, we applied forms that are commonly used in everyday speech, e.g., “The dog didn’t have any balls.” (In Hungarian, it is not possible to use a version that is close to the English “give no balls,” because, in Hungarian, the predicate is negated, and the results are similar to “Do not give none balls to the dog.”) In the “mathematical” linguistic version we used a form that reflects the mathematical viewpoint, e.g., “The dog had zero balls.”

One may ask whether the “natural” versions of these tasks really measure numerical abilities or, instead, measure some other abilities. For example, it is possible that the children used a non-numerical concept of nothing to solve the tasks instead of the numerical concept of zero. However, it is important to understand that, for a preschooler, both the concept of nothing and the concept of zero are appropriate to solve numerical tasks with zero. At their age, preschoolers can solve only a few numerical tasks: comparison, addition, and subtraction (Levine et al., 1992); in all of these tasks, both the concept of nothing and the concept of zero give the same correct result. Therefore, a correct or erroneous numerical task cannot differentiate in a simple way whether the concept of nothing or the concept of zero was used; consequently, the question of whether any of these concepts promote different strategies is not testable with the current methods.

In the analyses, the percentage of correct responses in the “zero,” “nothing,” and positive trials was used as the task’s performance index.

#### Smallest Number

The children were asked what the smallest number is. The aim of this task was to find out whether children regard zero as the smallest number and whether their performance on this task strongly correlates with the operations tasks (Aim 4). The task is similar to the task utilized in Wellman and Miller (1986). In the analysis, the given number was used directly. The task was applied as the first task of the session so that the children’s responses could not be influenced by other tasks, which might have taught them about the number zero (See further details in the order of the tasks part below). Furthermore, because several of the other tasks could affect this knowledge, we repeated this task at the end of the session to determine whether the children’ zero-knowledge had changed as a result of new knowledge they had acquired during the testing process.

#### Is It a Number?

The children had to categorize whether numbers as well as other things are numbers. The children were verbally asked, “Is the … a number?” The aim of this task was to study explicitly whether children regard zero as a number and whether their performance on this task strongly correlates with the operations tasks (Aim 4). To determine whether the children understood this categorization task, additional numerical and non-numerical words were used to validate the task. The following six words were tested in the given order: three, two, nothing, kitten, pop (sound), and zero. In the analysis, the given responses for the six trials were used directly. Similar to the smallest-number task, this task was presented at the beginning of the session, and it was repeated at the end of the session.

#### Order of the Tasks

Because some of the tasks could provide information about the meaning and use of the number zero to the children, we set the order of the tasks based on their potential to modify the responses of the children in later tasks. Therefore, the tasks concerning the status of zero were tested first. Additionally, since tasks including both verbal and object versions at the same time could teach the children the meaning of zero, we placed them at the end of the session. Finally, because the presented stimuli in the comparison, addition, and subtraction tasks depended on the number knowledge of the children, the give-N task preceded the comparison, addition, and subtraction tasks. Thus, the following order for the tasks was applied: smallest number, is-it-a-number, give-N task, comparison, verbal addition and subtraction, object addition and subtraction, smallest number (repeated), and is-it-a-number (repeated).

### Procedure

After receiving the written consent of the parents and verbal consent from the children themselves, the measurement took place in a separate room in a building at their preschool. Data collection for a single child took approximately 30 min, and required a single session (See the order of the tasks above at the end of the tasks section).

### Analysis Plan

To introduce the main contrasts and tests as they pertain to the tasks and aims, here, we provide a summary of the main analyses applied in the results section. Based on the give-N task, 2 × 2 categories are formed based on whether a child is a “zero”-giver or “zero”-non-giver and whether a child is a subset-knower or a CP-knower. In the comparison, addition, and subtraction tasks, (a) to see whether zero is more difficult to handle than positive numbers, correct response percentages are compared between tasks including zero and tasks including only positive numbers, (b) to investigate the effect of language, correct response percentages are contrasted in the verbal vs. non-verbal conditions and in the natural linguistic version vs. the mathematical linguistic version, and (c) to investigate the effect of number knowledge measured with the give-N task, correct response percentages are compared between subset-knowers and CP-knowers. In the smallest-number task, the distribution of the given responses are analyzed, and the frequencies of responses are compared between the different groups of number knowledge (i.e., subset-knowers vs. CP-knowers, and zero-givers vs. zero-non-givers). In the is-it-a-number task, correct response percentages are compared between non-numbers, positive numbers, and zero conditions, and this performance is compared between the groups of number knowledge (See more details on the specific analyses in the relevant results sub-sections).

## Results and Discussion

To make the results, their interpretation, and their connection to the specific aims easier to follow, this section groups the results by tasks and not by aims. Still, for all tasks, specific sets of their analyses are labeled by specific aims. In most task sections, the main results are described first; this is followed by relevant inferential statistics; and the section closes with the discussion of the results.

### Groups Based on Number Knowledge

#### Giving Positive Numbers

First, with the give-N task, we specified the number knowledge level of the children (subset-knowers vs. CP-knowers). This categorization was used in the following tasks to contrast the children based on their number knowledge. Twenty children understood the cardinality principle (i.e., they could solve the give-N tasks perfectly for the numbers between 2 and 5), while 20 children had not reached yet this phase, i.e., they were subset-knowers. Two children from the latter group could not solve the give-2-balls task, and because the number one was not measured in the task, they could either be one-knowers or pre-knowers. As we could not specify whether these two children were subset-knowers or pre-knowers, we excluded them from further analysis. Among the remaining 18 subset-knowers there were seven two-knowers, ten three-knowers and one four-knower.

#### Giving “Nothing” and “Zero”

Second, independent of the previous number-knowledge categorization, we specified whether the children could solve the tasks involving the natural version of zero (nothing-givers) and the mathematical version of zero (zero-givers). Practically all children (96%) understood the natural version of the give zero task (“do not give any balls”), while the mathematical version (“give zero balls”) proved to be more difficult (only 45% of the children could give “zero”). None of the nothing-givers or the zero-givers solved the task by adding zero accidentally for unknown numbers because zero was never given when a positive number was asked by the experimenter.

This difficulty with the mathematical version of zero could be rooted either in not knowing the word “zero,” or in the unusual and unnatural form of the task (i.e., giving something which is nothing). As for the former explanation, notes by the children show that at least some of them did not know what the word “zero” refers to; examples include, “What does zero mean?,” “I cannot count up to zero,” “That would be too much for me,” and “Zero is hundred.”

#### Relation of Giving Positive Numbers and Zero

Computationally, giving positive numbers and giving “nothing” or “zero” could be independent. The following analysis investigates whether empirical data support independence. The present data show that most children could solve the natural-zero task, independent of whether they were subset-knowers or CP-knowers (92% of subset-knowers and 100% of CP-knowers), meaning that giving “nothing” is independent of whether a child is a subset-knower or CP-knower. Additionally, while giving “zero” depends on their positive number knowledge (63% of CP-knowers and 25% of subset-knowers gave zero successfully) giving zero is not strictly connected to such knowledge: Not all CP-knowers could give zero, while some of the subset-knowers could. Note that this result is unlikely to be random noise. For example, in our sample, the CP-knowers could give all positive numbers (i.e., between 2 and 5) successfully on two occasions, but they were unable to do so successfully with “zero.” It is also noteworthy that even some of the subset-knowers understood the word “zero.” All of the subset-knowers solving the mathematical version of the give-zero task were “three”-knowers.

The previously described effects were analyzed with a 2 (subset-knower group vs. CP-knower group as a between-subjects factor) × 2 (“natural” vs. “mathematical” versions of the zero task as a within-subjects factor) ANOVA on the proportion of correct responses: A main effect of number knowledge [*F*(1, 36) = 7.59, *p* = 0.009, η*_*p*_*^2^ = 0.174, better performance in the CP-knower group], and a main effect of linguistic version [*F*(1, 36) = 46.62, *p* < 0.001, η*_*p*_*^2^ = 0.564, better performance with the “nothing” version] were found to be significant; a tendency in the interaction [*F*(1, 36) = 3.66, *p* = 0.064, η*_*p*_*^2^ = 0.092, CP-knowers were more successful in giving “zero” than the subset-knowers] was also found.

#### Groupings for the Following Analyses

Positive number knowledge (whether a child is a subset-knower or a CP-knower) is used throughout the following tasks to investigate whether this knowledge influences understanding zero (Aim 3). Because giving “zero” is independent of their positive number knowledge and because giving “zero” may reflect their ability to understand the label “zero,” the children were also categorized by this ability. A child was categorized as zero-giver, if the mathematical version (“zero”) of the task was solved correctly in both trials. We could not rely on the natural version (“nothing”) of the task because this task seemed to be trivial for preschoolers, resulting in a ceiling effect.

**FIGURE 1:**
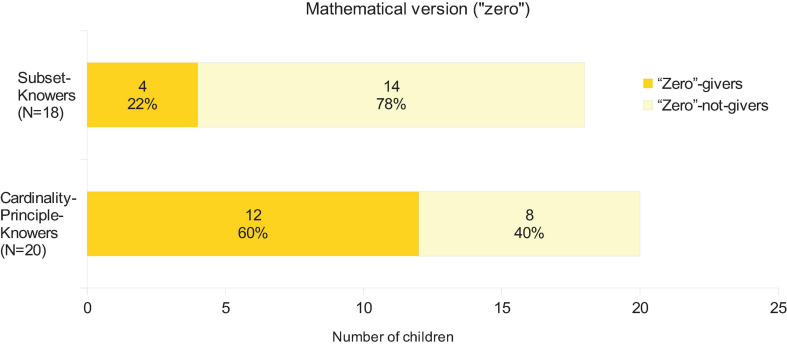
Number of the children who understood the mathematical (“giving zero”) version of the zero task.

These two orthogonal dimensions created four groups of children that were used in the subsequent analyses (Figure 1): 4 subset-knower and zero-giver (4.08 years mean age 0.26 SD), 14 subset-knower and zero-non-giver (3.8 years mean age, 0.4 SD), 12 CP-knower and zero-giver (4.33 years mean age, 0.55 SD), and 8 CP-knower and zero-non-giver (4.06 year mean age, 0.37 SD). A child is categorized as a zero-giver if they can correctly solve the mathematical versions of the give-zero task.

Note that, in the operations tasks, because of some missing data the subset-knower—zero-giver group is excluded, therefore, only 3 groups are compared. However, for the meta-knowledge tasks all 4 groups are analyzed. Also note that, because there may be an interaction effect in their positive number knowledge and giving zero, in the upcoming analyses, the 4 (or 3) groups will be handled as 4 (or 3) independent groups and not as 2 × 2 factors.

### Operations With Zero: Comparison, Addition, and Subtraction

These tasks investigated (1) whether the value of zero is more difficult to handle than positive integers, (2) the effect of language (a) contrasting verbal and non-verbal versions, which could reveal whether the children have either linguistic or conceptual problems with the zero value, and (b) contrasting the natural linguistic version (e.g., “add nothing to three”), and the mathematical linguistic version (e.g., “add zero to three”), and (3) the differences between CP-knowers and subset-knowers.

Because of a data collection problem, the data of 2 out of 4 subset-knower and zero-giver children’s data were not available in these tasks. Since the data of the 2 remaining children may be misleading due to the extremely small sample size, this whole group was excluded from the analysis.

**FIGURE 2:**
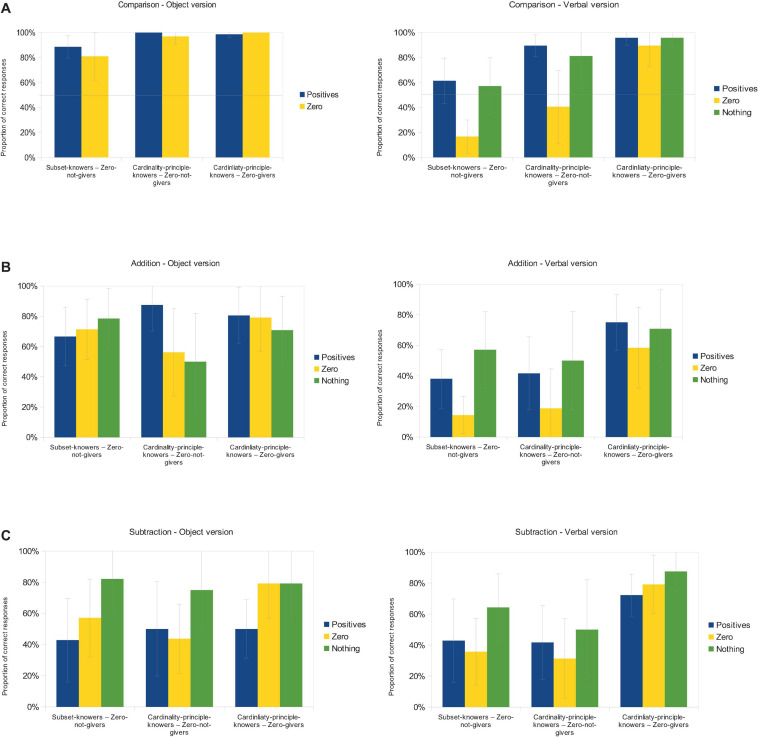
Proportion of correct responses in the comparison **(A)**, addition **(B)**, and subtraction **(C)** tasks in the non-verbal (left) and verbal (right) versions with positive integers, zero, and nothing (columns) within the different number-knowledge groups (categories on x axes). Error bars show 95% confidence interval. Horizontal line at 50% in the comparison task shows the random choice level.

The results are presented as population estimations of the mean correct response proportions (Figure 2) and hypothesis tests of those values (Table 2). Different analyses were run for the three tasks (comparison, addition and subtraction—see rows in Table 2) and for the object and verbal versions of the tasks (see columns in Table 2). Within these analyses, the number-knowledge groups (x axes on Figure 2; subset-knower and zero-non-giver, CP-knower and zero-giver, CP-knower and zero-non-giver) and number types (columns in Figure 2; positive values, zero, and nothing, except in the comparison object version, where distinguishing nothing and zero would not make sense) were used. Mixed ANOVAs applied number knowledge as a between-subject factor and number types as a within-subject factor.

**TABLE 2:** ANOVA results of the operations tasks.

	Object version	Verbal version
Comparison	**Number type main effect:** ns **Group main effect:** *F*(2, 31) = 4.13, *p* = 0.026, η*_*p*_*^2^ = 0.211; the subset-knowers show worse performance compared to the other two groups, *p*s < 0.037 **Interaction:** ns	**Number type main effect:***F*(2, 62) = 14.13, *p* < 0.001, η*_*p*_*^2^ = 0.313; the performance for “zero” was worse than for “nothing” or positives, both *p*s < 0.001 **Group main effect:** *F*(2,31) = 18.95, *p* < 0.001, η*_*p*_*^2^ = 0.55; all groups differed from each other, all *p*s < 0.017 **Interaction:** *F*(4, 62) = 2.45, *p* = 0.056, η*_*p*_*^2^ = 0.136
Addition	**Operand type main effect:** ns **Group main effect:** ns **Interaction:** ns	**Operand type main effect:***F*(2, 62) = 5.91, *p* = 0.004, η*_*p*_*^2^ = 0.016; tasks with “zero” were harder to solve than the two other task types, both *p*s < 0.02, LSD-test. **Group main effect:** *F*(2, 31) = 5.14, *p* = 0.012, η*_*p*_*^2^ = 0.249; CP-knower and zero-giver group shows better performance compared to the other two groups, both *p*s < 0.018. **Interaction:** ns
Subtraction	**Operand type main effect:***F*(2, 62) = 7.84, *p* = 0.001, η*_*p*_*^2^ = 0.202; the tasks with “nothing” were easier to solve than the two other task types, both *p*s < 0.003 **Group main effect:** ns **Interaction:** ns	**Operand type main effect:***F*(2, 62) = 3.13, *p* = 0.05, η*_*p*_*^2^ = 0.092; the tasks with “nothing” were easier to solve than the tasks with positives, or “zero,” both *p*s < 0.05 **Group main effect:** *F*(2, 31) = 5.92, *p* = 0.007, η*_*p*_*^2^ = 0.276; the CP-knower and zero-giver group shows a better performance compared to the other two groups, both *p*s < 0.007 **Interaction:** ns

*All analyses were run on the correct response ratios. The following designs were applied: in the object comparison version, 2 (number types: pairs with positive numbers vs. pairs with zero as a within-subjects factor) × 3 (number-knowledge groups as a between-subjects factor); in the verbal comparison version, 3 (number types: pairs with positive numbers vs. pairs with “zero” vs. pairs with “nothing” as a within-subjects factor) × 3 (number-knowledge groups as a between-subjects factor); in the addition and subtraction tasks, 3 (operand types: positive operands vs. zero vs. nothing as a within-subjects factor) × 3 (number-knowledge groups as a between-subjects factor). Post hoc tests are LSD tests. Note that, from the viewpoint of the current tests, the main effect of number or operand types and the interactions are relevant, but not the main effect of groups.*

#### Object Version

First, in the object versions of the tasks, overall, zero was not more difficult to handle than positive values (left in Figure 2; Aim 1): In the appropriate ANOVAs, the main effect of the number or operand type was not significant in the comparison and addition tasks, and the interaction was not significant in either of the tasks (left in Table 2).^3^ The only positive exception is that subtracting “nothing” was easier than subtracting positive numbers (a significant main effect of operand type in the object subtraction task; see Table 2), which is reasonable, since, in a relatively difficult subtraction task, it is easier to do nothing than to subtract a positive value. Thus, these results show that handling zero is not harder than other numbers for preschoolers in the object version of the tasks. This means that preschoolers understood the value of zero conceptually. This shows that comparison, addition and subtraction operations with zero are available as soon as one understands positive integers.

Note that the verbal variations of “nothing” and “zero” did not cause any significant effects (no main effect of number or operand type in comparison and addition tasks and no significant *post hoc* difference between those values in the subtraction task; see Table 2), which is understandable in these tasks where the verbal form could be complementary to the object based presentation (Aim 2). An early ability to handle zero as efficiently as positive numbers in these operations is true for both CP-knowers and subset-knowers as reflected in the same pattern in both groups (no interaction in any of the tasks; see Table 2; Aim 3).

#### Verbal Version

Second, in the purely verbal versions of the tasks, the zero value with the natural linguistic version (“nothing”) was not more difficult to handle than the positive values (right in Figure 2): No main effect of number or operand type in comparison or addition tasks that is caused by the difference of “nothing” and positive number performance (right in Table 2; note that the significant difference of the mathematical version (“zero”) is not a relevant contrast here). As in the case of the object version of the task, subtraction with “nothing” shows a positive exception, as it is easier than subtraction with positive numbers (significant main effect of operand type in the subtraction task; see Table 2). Again, these results show that understanding zero is available as soon as one understands positive integers (Aim 1).

However, handling zero values with the mathematical linguistic version (“zero”) revealed difficulties (significant main effect of number or operand type in all tasks; see Table 2; Aim 2). Importantly, this difficulty was seen mainly in the zero-non-giver groups, but not in the zero-giver groups (significant interaction in the comparison task, see Table 2; and see similar patterns in the population estimations in all of the three tasks, see Figure 2). This pattern means that in a trivial way, children who do not know what the word “zero” refers to cannot solve the tasks including that unknown word. This interpretation is confirmed by the fact that these tasks can be solved when the task is also supported by object demonstrations.

Finally, the relative difficulty of handling zero and the linguistic form did not differ between subset-knowers and CP-knowers, as reflected in the similar relative patterns across the number-knowledge groups (see the similar patterns in parameter estimations between the subset-knowers and zero-non-givers and the CP-knowers and zero-non-givers, Figure 2; Aim 3).

To summarize, the verbal version of the operations tasks also confirms that preschoolers can handle zero as efficiently as positive numbers, with the trivial exception of children who do not know what the word “zero” refers to. Since the same tasks were solved efficiently in the object task, it confirms that the difficulty was linguistic in nature and not conceptual. Again, no difference between subset-knowers and CP-knowers could be found in these contrasts.

#### Alternative Interpretations

Some alternative interpretations of these data could be considered. One may raise that, when solving addition or subtraction tasks with zero, children cannot solve the task and, instead, simply repeat the last operand, which would sometimes result in the correct solution, e.g., in the task 0 + 3, repeating the last operand is the correct result. However, our results are not in line with this possibility. First, if the children did not know zero and only used this mechanically incorrect solution, they should have given incorrect responses in the comparison task as well, which was not the case. One may think that the children could have used the appropriate solution in comparison, and it is only addition and subtraction where they used the incorrect method. Nonetheless, this account cannot explain that if the children know how addition and subtraction work (as seen in the positive operand tasks) and if they know how zero can be ordered relative to other values (as seen in the correct zero-comparison task), why they do not use these pieces of knowledge in a zero-arithmetic task. Second, in these tasks, this last-operand repeating strategy would give the correct result only in half of the present additions, e.g., for 0 + 3, the correct result can be given, while for 2 + 0, an incorrect result would be given, and none of the subtraction tasks can be solved, e.g., for 2–0 an incorrect result could be given (see the specific stimuli in the Tasks section). However, in the addition task, the addition performance is higher than the 50% performance that could be expected by this alternative interpretation. Still, one may think, that in those tasks, children do not use the last operand, but they use the known operand. However, in other tasks (e.g., the comparison tasks) the same children handled zero appropriately, so it is less likely that they do not know zero.

Another potential incorrect heuristics is that the children could compare the numbers instead of adding or subtracting them, which is a strategy that could result in correct responses in the tasks that include zero. However, this strategy would result in incorrect responses in the tasks that include only positive numbers, and such incorrect responses were not observed; thus, most preschoolers did not use this strategy either. Overall, if all tasks and results are considered together, one can conclude that the children handle zero on the same level as they handle positive numbers.

### Meta-Knowledge of Zero

#### Smallest Number

Most children thought that the smallest number was “one” (Table 3). Although some zero-givers proposed “zero” as the smallest number, even most of the zero-givers thought that the smallest number was “one.” None of the children proposed a negative number as an answer, although this happened once in one of our pilot studies.

**TABLE 3:** Proportion of different replies to “what is the smallest number?” in the four groups of number knowledge at the beginning of the session.

	Subset-knowers		Cardinality-principle-knowers	
	Zero-non-giver (*N* = 14)	Zero-giver (*N* = 4)	Zero-non-giver (*N* = 8)	Zero-giver (*N* = 12)
Zero	1 (7%)	1 (25%)		3 (25%)
One	2 (14%)	3 (75%)	6 (75%)	9 (75%)
Two	2 (14%)		1 (13%)	
Three	1 (7%)			
Five	1 (7%)			
Nothing	2 (14%)			
Does not know	5 (36%)		1 (13%)	

These statements are supported by a chi-squared test on the proportion of responses (0; 1; other numbers; nothing; does not know), which showed a significant effect [χ^2^(4, *N* = 38) = 26.47, *p* < 0.001], reflecting a high proportion of “one” responses. A chi-squared test on the responses between the four number-knowledge groups revealed a significant difference [χ^2^(12, *N* = 38) = 23.5, *p* = 0.024], which most probably reflects the heterogeneous responses in the subset-knower and zero-non-giver group and the relatively uniform “one” responses in the other groups.

At the end of the session (i.e., after solving all other tasks), the smallest-number task was repeated, with the answers basically the same pattern as at the beginning of the session.

When evaluating the possible dissociation of zero knowledge in the former comparison task and in the present smallest-number task (Aim 4), we should consider zero-givers (because zero-non-givers typically could not name zero) and CP-knowers (because, in the comparison task, we had no subset-knowers within the zero-givers). The smallest-number task is in clear contradiction with the comparison task: While in the comparison task, zero-giver CP-knower children knew that zero was smaller than one (83% correct response performance in the verbal version and 100% in the object version), they believed that one was the smallest number (only 25% of them believed that zero is the smallest number). This result repeats the pattern that can be seen in the data of Wellman and Miller (1986). One could argue that the children may have misunderstood the task. However, it is hard to imagine that they misunderstood the smallest-number task, since CP-knower children can understand conceptual properties of numbers, such as that number words for large numbers with an unknown position in the counting list are numbers (Lipton and Spelke, 2006), and they can understand how set size change runs parallel with the counting list steps (Sarnecka and Carey, 2008). A possible resolution for this result is that preschoolers believed that zero was not a number. The subsequent task more explicitly tested whether or not children regard zero as a number.

Contrasting the subset-knowers and the CP-knowers in this task (Aim 3), on the one hand, neither of these groups considered zero to be the smallest number. On the other hand, the subset-knowers gave more heterogeneous responses, which is in line with the fact that they experienced difficulties in solving the verbal comparison task (Figure 2). Overall, while the subset-knowers show a qualitatively different response in the smallest-number task compared to the CP-knowers, this difference is most probably related to their capability in comparison and not to their zero-knowledge.

#### Is It a Number?

**FIGURE 3:**
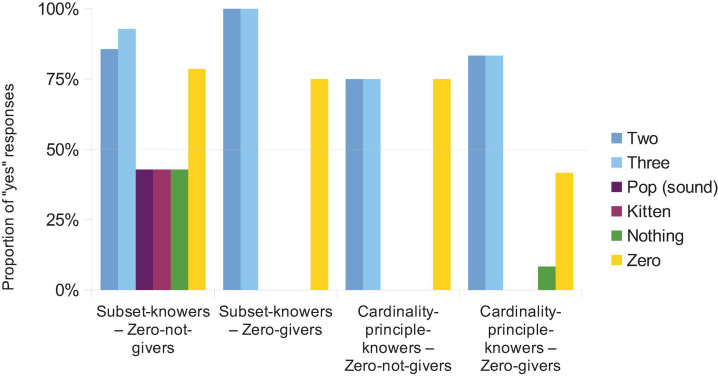
Proportion of “yes” responses in the is-it-a-number task at the beginning of the session. Horizontal line at 50% shows the random choice level.

Most groups thought that “two” and “three” were numbers, while “pop” (sound) and “kitten” were not numbers, although the subset-knower—zero-non-giver group showed an approximately random performance (Figure 3). This means that, except for the latter group, the preschoolers understood the task.

Critically, while the word “nothing” was not evaluated as a number, the status of “zero” is uncertain. This result is in line with the finding that even some of the adults question whether zero is a number (Wheeler and Feghali, 1983) and could be in line with our interpretation of the results from the smallest-number task, as described above.

Supporting these statements, a 4 (number-knowledge groups as a between-subjects factor) × 6 (words to evaluate as a within-subjects factor) ANOVA on the proportion of correct responses (not on the proportion of “yes” responses as displayed in Figure 3) revealed a main effect of groups [*F*(3, 34) = 3.008, *p* = 0.044, η*_*p*_*^2^ = 0.21], with the subset-knower and zero-non-giver group showing poorer performance compared to the other three groups (all *p*s < 0.037), and an interaction between the two factors [*F*(15, 170) = 2.12, *p* = 0.011, η*_*p*_*^2^ = 0.158]. To find the source of this interaction, a similar ANOVA was run, excluding the subset-knower and zero-non-giver group. The 3 (groups) × 6 (words) ANOVA revealed only a main effect of words [*F*(5, 105) = 3.34, *p* = 0.008, η*_*p*_*^2^ = 0.137]. *Post hoc* LSD-tests showed that the error rate for “zero” was higher than for “pop,” “kitten,” and “nothing.” Thus, in the three groups, evaluating “zero” was ambiguous, while the excluded subset-knower and zero-non-giver group showed random responses, reflecting that they did not understand the task.

Similar to the case of the smallest-number task, at the end of the session (after solving all other tasks), the is-it-a-number task was asked again, and the pattern of the responses did not change.

The results here show that, while the positive numbers are considered as numbers by the preschoolers, they do not think that the word “nothing” is a number, and they are ambiguous about the numerosity of the word “zero.” (Note that here we ignore the results of the subset-knower—zero-non-giver group because, according to the data, this group did not understand the task.) The ambiguity of the word “zero” is partly understandable in the zero-non-giver—CP-knower group, since they can only guess. However, the result is more informative in the zero-giver groups since they could use the zero value correctly in numerical tasks even when the number was labeled as “zero.” How can these differences be interpreted? There could be different factors that may influence children’s decisions about numberness. First, all these words could be and were used by the preschoolers appropriately in the numerical tasks (at least in the CP-knower and zero-giver group), thus, the difference between the number status of the words cannot be caused by the children’s numerical knowledge. Second, the word “nothing” can be used more generally in non-numerical cases and, additionally, unlike other number words, “nothing” cannot be used before nouns. These conceptual and linguistic differences may explain, why the word “nothing” is considered to be less numeric than the positive numbers and “zero.” Importantly, none of these numerical, conceptual and linguistic considerations could explain why the word “zero” is considered to be less numeric than the positive numbers. Thus, we argue that the results reflect that preschoolers do not regard “zero” as a typical number. (Although the word “nothing” is also considered to be less numeric than the positive numbers, one cannot tell how strongly the mentioned conceptual and linguistic viewpoints influenced this decision and, consequently, how strongly the real perceived number status of “nothing” influenced the decision.) This could mean that either (a) at least some of the children do not regard “zero” as a number, or (b) judging the numberness of a value on a continuous scale (e.g., prototypicality of the value as a number) “zero” is not considered as a typical number, or (c) most children are confused about the number status of zero, and simply guessed in this task. To summarize, this result is consistent with the idea that children do not think that zero is a typical number.

Regarding the connection between operational knowledge and meta-knowledge (Aim 4), the same difference can be observed here as in the smallest-number task: While these children (and especially in the zero-giver–CP-knower group that may be our main interest) can handle zero in numerical operations, they are uncertain whether zero is a number. This finding is again in line with the additional phase we emphasized in the data from Wellman and Miller (1986): At a specific point in their development, preschoolers can handle numerical operations with zero, although they do not regard zero as a typical number.

When contrasting the subset-knower and CP-knower groups, the only difference is that the subset-knowers seem to be unsure about the meaning of the task, and seemingly subset-knower–zero-non-giver children gave responses around the 50% random level for all categories.

### Reliability of the Data

Forty preschoolers participated in this study, who were categorized into four groups in the analyses, which resulted in rather small groups. Consequently, one may question how reliable our results can be. (1) Obviously, hypothesis tests control for small sample sizes: If the present results are significant, they are significant despite the small sample size. In other words, smaller samples need larger effect sizes to reach a significant result. This means that some of the current effects of interest are so large that even a relatively small sample is sufficient for a significant test or an appropriate power. (2) Moreover, a similar study was run before the present study. The present study is an improved version of our first measurement. The main differences between our previous and current measurements are that (a) the order of the tasks was modified, since, in the present version, all tasks that could potentially train the children were at the end of the session, (b) a few simple control tasks were removed, (c) there were minor improvements in the stimuli and instructions to have better control over the stimulus properties and the verbal conditions, and (d) the children were older, instead of 3- and 4-year-old children, 4- and 5-year old children participated. Importantly, the pattern of results was the same between the two studies: In the comparison and arithmetical tasks, zero was handled at the same level as positive numbers in both the subset-knower and CP-knower groups, even though the children were unsure about the number status of zero. Two notable differences that we found could readily be explained by the fact that the participants of the first measurement were older than the participants of the main measurement: The performance of the first measurement on the number status of zero was somewhat better than the performance of the sample in the present main study, and, in the pilot study, no zero-giver subset-knowers were found while there were only 6 subset-knowers out of the 36 participants. Overall, while single measurements could be unreliable (Open Science and Collaboration, 2015) and replications can be vital in obtaining reliable results, the present replication confirms that our findings are reliable. (3) Additionally, the smallest-number and is-it-a-number tasks were repeated within the session; as reported above, the results showed the same pattern, confirming again that the present results are reliable. (4) Moreover, the small size of the subset-knower, zero-giver group (4 children) cannot distort our results because their data in the operation tasks was not used at all, and almost all of our results rely on the data of the other three groups. (5) Finally, the results cannot be seen as a set of type-I and type-II errors or random noise because (a) similar and coherent patterns can be seen across independent tasks, (b) the results are coherent with many former reports, and (c) the results form a meaningful and coherent picture of the development of preschoolers’ understanding of zero. With such a large number of tasks and statistical analyses, it is highly improbable that a small sample and related random variation could form a coherent picture as observed here. Overall, these considerations strongly suggest that the present results are reliable and reveal real developmental patterns.

## General Discussion

Our results shed light on several important aspects of preschoolers’ understanding of zero. First of all, our data demonstrate that preschoolers understand the handling of empty sets in a numerical context and can appropriately apply it in various numerical tasks, such as giving a set with zero items, comparison, addition, and subtraction (Aim 1). Importantly, even subset-knowers can process empty sets appropriately: Their performance with empty sets was comparable with positive values if neither the mostly unfamiliar word “zero” nor the mathematical linguistic form was used (part of Aim 3). This result is in contrast with the results of Wellman and Miller (1986), who suggested that handling zero is difficult for preschoolers; this contradiction partly arises from the fact that, in that study, the children potentially could not understand the word “zero” and the mathematical linguistic form (see also the discussion of Aim 2 below). Additionally, while Wellman and Miller (1986) propose that children understand zero only when they know that zero is the smallest number, we argue that handling zero in numerical tasks is a sufficient criterion, while knowing that zero is the smallest number is knowledge that is irrelevant for evaluating preschoolers’ operational capabilities (see also the discussion of Aim 4 below).

Second, while preschoolers can handle empty sets, they have difficulties with mathematical language (Aim 2). One component of this linguistic difficulty is the knowledge about the meaning of the word “zero.” The children who did not know the word “zero” could not solve the tasks in which the number zero was denoted purely by this label, while they could solve the same task with different wording, suggesting that the difference is whether they know that the “zero” label refers to a concept that they can otherwise use. Relatedly, some children explicitly noted that they do not know the meaning of the word “zero.” As a second linguistic component, the children had difficulty with the “mathematical” formulation of the tasks (e.g., “give zero balls to the bird”). Importantly, they could solve the task if (a) zero was denoted non-verbally or (b) the natural linguistic form of the zero-related statements (e.g., “do not give any balls to the bird”) was used. This means that again, the difficulties described here are linguistic, not conceptual, in nature. It is not surprising that the children had problems with the mathematical formulations since this form is mostly used for mathematical and formal purposes, and they had probably rarely heard it before. Our results also showed that these language-related difficulties could be observed independent of whether a child is a subset-knower or a CP-knower (part of Aim 3).

Third, the children had problems with the number status of zero (Aim 4). First, they were unsure whether zero is a number. Second, they exhibited a contradiction: They thought that 0 is smaller than 1, but they thought that 1 is the smallest number (the same contradiction was apparent in the study of Wellman and Miller, 1986). This contradiction can be seen as another reflection of being unsure whether zero is a number: While 0 is smaller than 1 (nothing is less than something), 1 is the smallest number because 0 is not a number. Again, this pattern was independent of whether a child is a subset-knower or a CP-knower, although the subset-knowers had some difficulty in understanding the meta-knowledge tasks.

Note that, while the present study did not measure detailed sociodemographic characteristics, former numerical training, or other related properties that may qualify the present results, when comparable, our results are in line with the results of previous studies. These correspondences suggest that the present findings are at least robust.

Overall, these results identify several more or less independent components of the zero-knowledge. The first component is operational knowledge, i.e., whether children can use zero in tasks, such as giving a set of objects, comparing values, or adding and subtracting numbers. The second component is linguistic knowledge, including whether children know that the label “zero” is used to denote an empty set and whether they know the specific (and somewhat contradictory) mathematical formulation (e.g., “add zero balls to the rabbit”). The third component is meta-knowledge: Whether children know that zero is a number. The present results demonstrated that these three components do not necessarily appear at the same time. Our data also demonstrate that these pieces of knowledge do not depend strictly on whether a child is a subset-knower or a CP-knower.

### Theoretical Account of the Development of Zero-Knowledge

What representations or accounts can explain this more detailed picture of the development of preschoolers’ understanding of zero? First of all, the linguistic effect is not surprising in the sense that linguistic knowledge of zero has an effect on how zero is handled in verbal tasks. From a theoretical viewpoint, what is more interesting is the different development of operational and meta-knowledge of zero. How is that children who do not think that zero is a typical number still can solve numerical tasks with zero correctly? Several explanations offer a solution to this problem.

A group of explanations may suggest that linguistic or cultural factors cause the difference between zero and positive integers in some tasks and, relatedly, they explain why operational and meta-knowledge of zero dissociate. However, we argue that these explanations cannot account for the present results. (1) An explanation posits that the special status of zero is due to its infrequent use in everyday language (Dehaene and Mehler, 1992). However, after understanding the cardinality principle children understand some common properties of numbers, even if that number is beyond the limit of their counting list (Lipton and Spelke, 2006). Critically, the frequencies of such numbers are comparable to the frequency of zero. Consequently, the relatively low frequency of the word “zero” cannot explain why children do not regard zero as a number, because, while a rare number (i.e., zero) is not regarded as number, other rare numbers (e.g., 20 and 40) are regarded as numbers. (2) Another example of a linguistic explanation posits that zero is categorized incorrectly because empty sets are handled linguistically in a different way than positive numbers: The word “nothing” is commonly used instead of “zero” and “natural” sentences are used instead of the “mathematical” versions. However, zero-givers (those who are familiar with these linguistic forms) are as unsure of the number status of zero as zero-non-givers; therefore, even if these rare linguistic forms are known, zero still can be thought as a non-number. Additionally, in a similar case of the number one, although children learn the distinction between one and many (i.e., other positive numbers) grammatically, which helps them to learn the meaning of “one” (Carey, 2009), they still think that one is a number. Thus, in the case of this linguistic difference, the relevant value (i.e., one) is not categorized differently than larger natural numbers. (3) A final possible linguistic-cultural explanation proposes that a counting list usually starts with one, which seemingly excludes zero from the set of numbers (Merritt and Brannon, 2013). However, at least in some cases, this issue is not rooted in the counting list. For example, some adults also think that zero is not a number, and their justifications are not related to the counting list: They say that zero is not a number because “a number is the abstract value of the quantity of a set” or because “it has no value” (Wheeler and Feghali, 1983). Additionally, it is unclear why this linguistic phenomenon would have an effect on categorization if the previous and potentially stronger linguistic effects (i.e., the first and second points of this paragraph) do not play a role in the categorization of zero. Importantly, none of these linguistic-cultural explanations can account for the fact that zero is handled differently only in some tasks, while it is handled similarly to positive integers in other tasks. To conclude, although we cannot entirely exclude all linguistic or cultural influences, these explanations cannot fully account for the dual nature of zero.

A second group of explanations supposes that there are representational causes why zero is not regarded as a typical number. (1) One can imagine that the representation supporting positive values cannot store the value of zero; hence, zero must be stored in a different system. The most frequently referred-to model suggests that semantic numerical processing is driven by the Approximate Number System (ANS) which represents numbers in an imprecise way (Moyer and Landauer, 1967; Feigenson et al., 2004; Merritt and Brannon, 2013). This system may include zero, although its ability to do so is debated (Dehaene et al., 1993; Dakin et al., 2011; Merritt and Brannon, 2013; Ramirez-Cardenas et al., 2016) (See more details on this system in Supplementary Material). Some part of the results could readily be explained by this model: In the operation tasks, even if the stimuli were presented symbolically, the imprecise representation may be handling the relatively small values used in the present experiment. However, it is not trivial to consider how the model accounts for the dual nature of zero. In the ANS framework, one may assume that while the positive numbers and, in some cases, zero value could be handled by this system (as in the comparison task), in some other cases, the zero value should be handled by another system (as in the number-status-of-zero task). However, it is not clear what this alternative system could be, and why the ANS sometimes could not handle zero. In other words, these suppositions are arbitrary, and the additional details were only created to account for these new results, while they are not supported by any other phenomena. Overall, it is hard to provide a coherent explanation of how the ANS could account for the special status of zero. (2) Another possible explanation suggests that understanding numbers relies on a conceptual understanding of the items (e.g., objects) in a set. For example, Carey (2004, 2009) proposes that children induce a conceptual understanding of how counting can specify the size of a set, which relies on set-templates in long-term memory; such templates are based on the activation of visual indexes. (It is important to note that it is not simply the visual index or Object Tracking System that supports the cardinality principle, because the visual index in itself is only a set of spatial indexes, which computationally is not capable of supporting conceptual understanding. Instead, some additional mechanisms, such as set-templates in the long-term memory, and other unspecified mechanisms are necessary to support a conceptual understanding of the cardinality principle.) Another model for explaining why sign-value notation numbers (e.g., Roman notation) are easier to understand than place-value numbers (e.g., Indo-Arabic notation) proposes that sign-value notations are more similar to a hypothesized item-based number representation in which the powers (e.g., ones, tens, hundreds, etc. in a 10-based system) are denoted by objects or groups (Krajcsi and Szabó, 2012). In both of these latter models, numbers could be considered as the properties of items or objects in a set; since there are no items or objects in an empty set, it would not make sense to talk about the property of the non-existing items, and, accordingly, zero could not be a number in this framework. The case of a property for a missing item is similar to the paradox introduced by Lewis Carroll: In his story, a smiling cat disappears, though the smile of the cat remains visible. In a similar way, the property (in this case, the numerosity) of the non-existent items is not meaningful, and the lack of items cannot be described in a similar way to a set of items. This model is also in line with the justifications provided by adults for why they do not regard zero as a number, i.e., they often state that numbers describe the items in a set (Wheeler and Feghali, 1983). Importantly, this view can explain why zero has a dual nature: Even though empty sets can be handled in numerical tasks that involve manipulating objects or items, the status of zero is special since it cannot represent the property of items that are not present, but it represents the lack of those items. Therefore, we conclude that a conceptual understanding of numbers as items or objects can explain why zero is handled in a special way.

### Implications for Practice

The present study has some educational consequences. Teaching and understanding the properties of zero is difficult (Lichtenberg, 1972; Frobisher, 1999). Understanding some simple properties, such as the parity of zero and the number status of zero, can be problematic, even for mathematics teachers (Wheeler and Feghali, 1983; Hill et al., 2008).

Based on our results, it is possible to offer some educational recommendations for schools and even preschools. At the same time, the efficiency of these recommendations may also validate and test our description of the zero-knowledge development. Our recommendations are based on the fact that several independent components of zero-knowledge can be identified (namely, operational knowledge, linguistic knowledge and meta-knowledge) and that such components develop at different paces. (1) Based on the present results for operational knowledge, children could handle empty sets as soon as they can handle positive numbers in basic numerical operations; thus, no further effort is needed to introduce this concept to them. (2) However, they are likely unfamiliar with the mathematical linguistic formulations of the problem; thus, one can teach them these linguistic forms. (a) One linguistic component to teach is that the word “zero” means “nothing” or “doing nothing” in mathematical problem-solving. (b) Another linguistic component to teach is the mathematical form of sentences; thus, one can recite both the “natural” and “mathematical” versions of the task, stressing that the two versions refer to the same thing, e.g., “do not give any balls to Suzy Sheep; you can also say, give zero balls to Suzy Sheep.” (3) Additionally, children do not understand the number status of zero, and they are unsure whether zero is a number. According to one possibility, one can explain to children that zero is also a number because we can use it in counting and other mathematical tasks. However, this solution raises a problem. If our explanation for the present results is correct, then children are not sure whether zero is a number because they consider that a number is a property of items, and zero describes the lack of items, which is a quantitatively different state. This “no items” state can handle empty sets in numerical tasks correctly, although it is not regarded as a number. Importantly, before school (and, in most educational systems, also in the first years of school), children have no experience that could demonstrate to them that the “no items” state is actually a number. For example, most adults know that zero is a number because it is simply another step on the number line between positive and negative integers or because, in written multi-digit calculations, zero is handled in a similar way as other digits. However, preschoolers do not have any experience that could reveal the numerical nature of the “no items” state because, for example, they do not know negative integers, or they cannot make multi-digit calculations. Thus, although one can explain that zero is a number because we use it in numerical tasks, this information still cannot be justified based on their experiences. Consequently, it seems more reasonable that it is unnecessary to provide any education on this issue before school, and it may be better to teach children about the number status of zero only when this information can be justified and can be built up in a reasonable way. To sum up, (1) preschoolers already know how to handle empty sets in basic numerical operations, although (2) mathematical language of referring to empty sets can be taught, while (3) teaching the number status of zero is probably unnecessary at this stage of their development.

## Conclusion

In conclusion, preschoolers can handle zero on the same level as they can handle positive integers. Although the linguistic form can cause difficulties for them, this is independent of their conceptual understanding. However, preschoolers are unsure whether zero is a number. This may be caused by the set-based representation of numbers: Numbers can be the properties of items in a set, and, since an empty set does not include any items, zero cannot be a number in this view.

## Data Availability Statement

The raw data supporting the conclusions of this article will be made available by the authors, without undue reservation.

## Ethics Statement

The studies involving human participants were reviewed and approved by ELTE Eötvös Loránd University, Department of Cognitive Psychology. Written informed consent to participate in this study was provided by the participants’ legal guardian/next of kin.

## Author Contributions

All authors contributed to the design of the study, analysis of the data, and writing the manuscript.

## Conflict of Interest

The authors declare that the research was conducted in the absence of any commercial or financial relationships that could be construed as a potential conflict of interest.

## Publisher’s Note

All claims expressed in this article are solely those of the authors and do not necessarily represent those of their affiliated organizations, or those of the publisher, the editors and the reviewers. Any product that may be evaluated in this article, or claim that may be made by its manufacturer, is not guaranteed or endorsed by the publisher.

## Footnotes

1 There are a few other related former works that are not relevant from the viewpoint of the present work. First, some of the works cited in the main text also investigate children in their first school years. Still, we mostly focus on the preschool years, as our study investigates knowledge of zero in preschoolers around the time when the cardinality principle is acquired. Second, while several former works investigated the understanding of zero in preschoolers, they study other aspects of this understanding not in line with the aims of the present study, and their results are not conclusive for the present aims. While Merritt and Brannon (2013) collect preschooler data about processing zero, the comparison task is not symbolic. Also, while Davidson (1992) uses numerical tasks with zero in preschoolers, those tasks alternate training tasks and questions, so it is not clear what is the effect of the training in the session. Finally, while Baroody et al. (2009) partly investigate symbolic arithmetical operations with zero, they do not report the results of positive number-only tasks; therefore, it is impossible to tell whether the children had more difficulty with zero than with positive numbers, and they measure the number knowledge of the children with the Is it N task, which is considered to be an invalid measure of the number knowledge by the literature (Wynn, 1990, 1992). Another seemingly related topic is the handling of zero in transcoding tasks. In transcoding tasks, one has to translate a number from a notation to another notation, e.g., read aloud an Indo-Arabic number, where Indo-Arabic notation is transcoded into number word notation. It has been demonstrated that zero digits are often transcoded erroneously, e.g., “two hundred and two” is transcoded into 2002 (Grana et al., 2003; Zuber et al., 2009; Moeller et al., 2015). While this phenomena is related to the present topic in the sense that it is related to zero, there are at least three critical differences. First, while the present work discusses the zero *number* (i.e., the value between 1 and −1), transcoding considers the zero *digit* (i.e., the symbol that is used to denote a missing power in multi-power notation; e.g., the number 10 is a number between 9 and 11, and the zero digit is used only to denote that in a decimal system that a number does not include ones). Second, place-value notational systems, such as the Indo-Arabic number system, are relatively difficult to understand both for adults and children (Krajcsi and Szabó, 2012), and transcoding tasks require not only an understanding of zero, but also an understanding of place-value and other multi-power notational systems. Consequently, it is not straightforward how strongly transcoding issues are rooted in zero digits handling or in multi-power notation processing. Third, syntactic processes behind transcoding are only relevant in multi-power (e.g., multi-digit Indo-Arabic) numbers that are typically not understood by preschoolers, but only by older children (Zuber et al., 2009; Moeller et al., 2015).

2 Number knowledge as measured with the give-N task can be calculated with several methods. Here, we consider two alternative evaluation methods, and report that these methods gave the same results as the method we reported in the main text. (1) In most published works, a number is judged to be known if the correct response rate is not lower than 66%; here, we used the 100% threshold value. Because we asked all numbers twice, the correct response could be only 100, 50, or 0%. The 100% criterion would underestimate the child’s number knowledge compared to the usual 66% criterion, while the 50% criterion would overestimate it. Because the correct response rate changes rapidly around the limit of the child’s knowledge, the 100 and 50% percent criteria give similar results. Given our analysis and exclusions, in most of our analysis the 50% criterion categorizes only two children as CP-knowers who were subset-knowers with the 100% criterion. We reran all analyses with both criteria, and they gave the same pattern of significant and non-significant results. For the sake of simplicity, we only present the results with the 100% criterion analysis here. (2) We also used an alternative Bayesian calculation method to specify whether someone is a subset-knower or a CP-knower (Negen et al., 2011), although, to our knowledge, the validity of this method has not been tested so far. It gave us a categorization result that was between the results of the previous 100 and 50% methods, thus, the results of this categorization are not presented here either.

3 In the figures and in the appropriate ANOVAs the critical information is the main effect of number type (positive numbers vs. zero vs. nothing), reflecting an overall effect of zero, or the interaction between number type and number-knowledge groups, reflecting a group specific effect of zero. However, the main effect of groups is not relevant because (a) differences between the groups only show whether some groups perform better in general and not the relative difference between zero and positive integer-related performance, and (b) the CP-knower and subset-knower groups received different tasks (see section “Materials and Methods”).

## Appendix

**TABLE A1:** The questions in all tasks, in Hungarian and in their English translations.

Hungarian version	English translation	English translation reflecting the Hungarian structure more strictly
**Give-N task**		
Non-zero version: Adj a madárnak … golyót.	Give … balls to the bird.	Give to the bird … ball (*singular*).
Natural zero version: A madárnak semennyi golyót se adj.	Do not give any balls to the bird.	To the bird none ball (*singular*) do not give.
Mathematical zero version: Adj a madárnak nulla golyót.	Give zero balls to the bird.	Give to the bird zero ball (*singular*).
**Comparison**		
Object version: Melyik oldalon van több?	On which side is there more?	On which side is there more?
Verbal version: Melyik a több, a … vagy a …?	Which one is more, the … or the …?	Which is the more, the … or the …?
**Addition and subtraction**		
Addition: Mennyi golyójuk van összesen, ha együtt játszanak?	How many balls do they have altogether when they play together?	How many ball (*singular*) they have altogether, if together they play?
Subtraction: Mennyi golyó marad a madárnak?	How many balls was the bird left with?	How many ball (*singular*) has left for the bird?
**Smallest number**		
Melyik a legkisebb szám, mi a legkevesebb?	What is the smallest number, what is the fewest one?	Which is the smallest number, what is the fewest?
**Is it a number?**		
A … az szám?	Is the … a number?	The …, is it a number?
