# Supplementary material for: Development of Preschoolers’ Understanding of Zero
Source: Front Psychol. 2021 Jul 27;12:583734. doi: 10.3389/fpsyg.2021.583734 (PMC8353124; doi:10.3389/fpsyg.2021.583734)
Supplement: Supplementary file 1 [file Table_1.doc]

# Supplementary material – How can infants handle zero?

In the infant literature, there is agreement that nonsymbolic numerical information (such as arrays of dots, and series of sounds or events) is probably processed by two representations.[[1]](#footnote-2) (1) The Approximate Number System (ANS) can represent numbers in an imprecise way. This number-specific representation stores the values noisily: The larger the value is, the noisier the representation is. This simple representation implements Weber’s law, i.e., in its general form, the performance of a number comparison task depends on the ratio of the two values: The larger the ratios of the values are, the better the performance is. In line with this law, it was found that infants are able to discriminate two values if the difference is larger than a specific ratio (Feigenson et al., 2004); this ratio (i.e., the Weber-fraction) improves with age (Piazza, 2010); therefore, the ANS is a representation that supports number-processing performance in infants. Note that, in recent years, there is an increasing body of evidence questioning the supposed role of the ANS in number processing, e.g., whether, in nonsymbolic tasks such as a dot comparison task, it is really the numerical property that influences performance or, alternatively, correlating visual properties have a strong effect on performance (Gebuis & Reynvoet, 2012), or, in an alternative model, symbolic number processing effects can be explained by a simple mechanism other than the ANS (Krajcsi et al., 2016, in press). (2) As another system to account for infants’ numerical performance, there is an Object Tracking System (OTS) or visual indexes that can represent objects up to a limit of 3 or 4 items; this system can implicitly store the number of such objects, and it is argued that some of the numerical performance of infants can be explained with this representation (Feigenson et al., 2004). While a consensus in the infant literature is that the ANS and the visual index systems may be responsible for the numerical performance observed in infants, it is not clear whether these systems can represent zero.

Regarding the ANS, Merritt and Brannon (2013) investigated whether four-year-old preschoolers can handle zero in a nonsymbolic comparison task. (Note that, although their work studied preschoolers, not infants, it directly investigates the role of the ANS in nonsymbolic number processing, which is similar to the number processing observed in infants.) They were looking for the distance effect (i.e., increasing error rate with smaller distance); this effect is considered to be a sign of ratio-based ANS processing (Moyer & Landauer, 1967). In the whole sample, the distance effect was observed in the comparison task, suggesting the role of the ANS in handling zero; however, other aspects of the results suggest that it was not the ANS that processed zero. First, many children could not solve the task: Children who performed relatively poorly in the positive-number comparison task performed randomly and did not show any distance effect with number pairs that included zero. However, poor performance for positive numbers is surprising because even much younger infants are sensitive to numerical information (Feigenson et al., 2004). Second, the performance with pairs that included zero was worse than with pairs including one; however, the ANS would predict better performance with the zero-pairs. The authors hypothesize that preschoolers may initially avoid zero because, for example, zero is not included in the usual counting sequence (Merritt & Brannon, 2013). Because it is assumed that the same ANS works in non-humans and humans across various ages with basically the same properties (Piazza, 2010), other studies may help us to specify whether the ANS can store zero. Unfortunately, it is not easy to determine whether the ANS can represent zero, even when investigated in adults or non-humans (Dehaene et al., 1993; Fischer & Rottmann, 2005; Merritt et al., 2009; Varma & Schwartz, 2011). One problem is that presenting a set with zero involves methodological issues, e.g., in the stimuli, nothing is shown, which provides radically different perceptual properties compared to non-zero stimuli. A related problem is that presenting nonsymbolic empty sets opens up different interpretational possibilities about what information can be used to differentiate empty sets from non-empty sets. Overall, the results for whether ANS may represent zero are inconclusive. While some authors argue that the ANS can store zero (Merritt & Brannon, 2013), these results are debatable; moreover,according to some models it would be impossible for the ANS to store zero (Wynn & Chiang, 1998) because, for example, the ANS may rely on perceptual properties, and missing stimuli (i.e., a nonsymbolic empty set) cannot activate the appropriate features in the system (Dakin et al., 2011). It is also possible that, even if zero is stored by the ANS, it requires additional supporting processes. For example, it was found that monkeys can solve a number-matching task with an empty set, and cells tuned to the empty sets were found both in the ventral intraparietal area and in the prefrontal cortex, as has been observed for positive numbers (Ramirez-Cardenas et al., 2016). However, while the cells in the prefrontal cortex showed the distance effect for zero, which is a sign of ANS processing, the intraparietal cells did not, even though the ANS is supposed to be localized in the intraparietal area (Dehaene et al., 2003). Thus, it is not clear whether the ANS can handle zero either in general or, specifically, in infants or preschoolers.

Some studies have investigated more directly whether infants can represent the lack of objects. Six- and 8-month-old infants seemingly cannot maintain the representation of the lack of an object as measured with violation-of-expectation paradigms: While they see the unexpected or “magical” disappearance of an object as an unexpected event, they do not see the “magical” appearance of an object as an unexpected event (Kaufman et al., 2003; Wynn & Chiang, 1998). On the one hand, we can assume that representing the lack of objects is handled by the representation that handles numerical information. In this case, because the numerical handling of small (i.e., less than 4 or 5 items) sets of objects is attributed to the visual indexes or to the OTS by default, and the ANS handles these small sets only in exceptional cases (Feigenson et al., 2004), the failure to detect a “magical” appearance supports the idea that it is the visual index or the OTS that cannot represent the lack of objects. On the other hand, it is also possible that representing the lack of objects is handled by some other systems, representing other aspects of objects or locations, and these results cannot specify whether the ANS or the visual index can handle empty sets. To summarize, there is no consensus on whether either the ANS or the visual index can represent zero, and there is only scant data describing whether these or other systems can handle zero in infants.

# References

Dakin, S. C., Tibber, M. S., Greenwood, J. A., Kingdom, F. A. A., & Morgan, M. J. (2011). A common visual metric for approximate number and density. *Proceedings of the National Academy of Sciences*, *108*(49), 19552–19557. https://doi.org/10.1073/pnas.1113195108

Dehaene, S., Bossini, S., & Giraux, P. (1993). The mental representation of parity and mental number magnitude. *Journal of Experimental Psychology: General*, *122*, 371–396. https://doi.org/10.1037/0096-3445.122.3.371

Dehaene, S., Piazza, M., Pinel, P., & Cohen, L. (2003). Three parietal circuits for number processing. *Cognitive Neuropsychology*, *20*, 487–506.

Feigenson, L., Dehaene, S., & Spelke, E. S. (2004). Core systems of number. *Trends in Cognitive Sciences*, *8*, 307–314.

Fischer, M. H., & Rottmann, J. (2005). Do negative numbers have a place on the mental number line? *Psychology Science*, *47*(1), 22–32.

Gebuis, T., & Reynvoet, B. (2012). The interplay between nonsymbolic number and its continuous visual properties. *Journal of Experimental Psychology: General*, *141*(4), 642–648. https://doi.org/10.1037/a0026218

Kaufman, J., Csibra, G., & Johnson, M. H. (2003). Representing occluded objects in the human infant brain. *Proceedings of the Royal Society of London. Series B: Biological Sciences*, *270*(Suppl 2), S140–S143. https://doi.org/10.1098/rsbl.2003.0067

Krajcsi, A., Kojouharova, P., & Lengyel, G. (in press). *Processing symbolic numbers: The example of distance and size effects*. https://psyarxiv.com/5wzcx

Krajcsi, A., Lengyel, G., & Kojouharova, P. (2016). The Source of the Symbolic Numerical Distance and Size Effects. *Frontiers in Psychology*, *7*. https://doi.org/10.3389/fpsyg.2016.01795

Merritt, D. J., & Brannon, E. M. (2013). Nothing to it: Precursors to a zero concept in preschoolers. *Behavioural Processes*, *93*, 91–97. https://doi.org/10.1016/j.beproc.2012.11.001

Merritt, D. J., Rugani, R., & Brannon, E. M. (2009). Empty sets as part of the numerical continuum: Conceptual precursors to the zero concept in rhesus monkeys. *Journal of Experimental Psychology: General*, *138*(2), 258–269. https://doi.org/10.1037/a0015231

Moyer, R. S., & Landauer, T. K. (1967). Time required for Judgements of Numerical Inequality. *Nature*, *215*(5109), 1519–1520. https://doi.org/10.1038/2151519a0

Piazza, M. (2010). Neurocognitive start-up tools for symbolic number representations. *Trends in Cognitive Sciences*, *14*(12), 542–551. https://doi.org/10.1016/j.tics.2010.09.008

Ramirez-Cardenas, A., Moskaleva, M., & Nieder, A. (2016). Neuronal Representation of Numerosity Zero in the Primate Parieto-Frontal Number Network. *Current Biology*, *26*(10), 1285–1294. https://doi.org/10.1016/j.cub.2016.03.052

Varma, S., & Schwartz, D. L. (2011). The mental representation of integers: An abstract-to-concrete shift in the understanding of mathematical concepts. *Cognition*, *121*(3), 363–385. https://doi.org/10.1016/j.cognition.2011.08.005

Wynn, K., & Chiang, W.-C. (1998). Limits to Infants’ Knowledge of Objects: The Case of Magical Appearance. *Psychological Science*, *9*(6), 448–455. https://doi.org/10.1111/1467-9280.00084

1. Although the present work does not investigate the numerical abilities of infants, the preschooler numerical processing models rely on these infant models, and they are relevant in the interpretation of the current results. [↑](#footnote-ref-2)
